# Implementation of an acute DVT ambulatory care pathway in a large urban centre: current challenges and future opportunities

**DOI:** 10.1186/s12959-019-0203-y

**Published:** 2019-07-10

**Authors:** Sarah Kelliher, Patricia Hall, Barry Kevane, Daniela Dinu, Karl Ewins, Peter MacMahon, Fionnuala Ní Áinle, Tomás Breslin

**Affiliations:** 1 Department of Haematology, Mater Misericordiae University Hospital, Eccles St, Dublin 7, Ireland; 2 Department of Emergency Medicine, Mater Misericordiae University Hospital, Eccles St, Dublin 7, Ireland; 3 Department of Haematology, Rotunda Hospital, Dublin 1, Ireland; 4 Department of Radiology, Mater Misericordiae University Hospital, Eccles St, Dublin 7, Ireland; 5 School of Medicine, University College Dublin (UCD), Dublin 4, Ireland; 6 UCD Conway Institute SPHERE Research Group, UCD, Dublin 4, Ireland

**Keywords:** Venous thromboembolism, Deep venous thrombosis, Anticoagulation, Direct oral anticoagulant, Ambulatory care pathway, Intravenous drug use

## Abstract

**Background:**

Ambulatory management of isolated acute deep venous thrombosis (DVT) is the recommended standard of care in selected populations. However, in practice a significant number of patients continue to be managed as in-patients.

**Objectives:**

In this study we aimed to evaluate acute DVT treatment pathways in our emergency department (ED) in practice and to identify barriers to outpatient management.

**Methods:**

This study was a cross-sectional analysis of prospectively collected data pertaining to consecutive patients presenting to the ED of a large, city center, academic teaching hospital over a 46 week period who were diagnosed with DVT.

**Results:**

Implementation of an outpatient care pathway led to the majority of patients presenting with DVT in our institution being treated without hospital admission. Forty percent (31/78) of patients with DVT were treated with a direct oral anticoagulant (DOAC) as an outpatient in line with international best practice guidelines.

**Conclusion:**

The study provides a clear picture of the clinical profile and management of patients in clinical practice. Due to the lack of resources and supported infrastructure it is difficult to effectively implement outpatient venous thromboembolism (VTE) management to its full potential. Directing resources towards strategies which facilitate outpatient DVT treatment among vulnerable patient groups could represent a means of reducing hospital admissions for DVT in urban centers. Our study highlights the success and clinical limitations of the outpatient treatment model, which should become standard as part of wider VTE care.

## Introduction

Venous thromboembolism (VTE) comprises deep vein thrombosis (DVT) and pulmonary embolism (PE) and is a major contributor to global disease burden, affecting millions of individuals worldwide every year [1, 2]. The incidence of DVT in Europe has recently been reported as 70–149 cases/100000 person-years [3]. The majority of cases are diagnosed and initial treatment is commenced in the emergency department (ED) [4, 5]. It has been established that ambulatory management of isolated DVT, without inpatient admission, is safe and feasible in appropriately selected populations [6–9]. Currently published data suggests that ambulatory care with low molecular weight heparin (LMWH) is safe for selected patients with acute DVT [7]. In recent years, direct oral anticoagulants (DOACs) have been compared with warfarin in randomized phase 3 trials and are now suggested as first-line treatment for VTE over vitamin K antagonists for most patients [10]. Moreover, DOACs are associated with additional benefits in particular for patient management in the outpatient setting, including lack of requirement for monitoring of anticoagulant effect and a lower risk of major haemorrhage including intracranial haemorrhage [11, 12]. International guidelines from the European Society of Cardiology recommend the application of specific clinical criteria to identify patients with DVT suitable for outpatient or ambulatory management [13]. However despite this available evidence, in practice a significant number of patients (> 50%) continue to be managed as in-patients, including those potentially suitable for out of hospital management [4, 12, 14]. In this study we aimed to evaluate acute DVT treatment pathways in our ED, to examine the implementation of these guidelines in practice and to identify barriers to outpatient management.

## Methods

This study was a cross-sectional analysis of prospectively collected data pertaining to consecutive patients presenting to the emergency department (ED) of a large, city centre, academic teaching hospital in Dublin City Centre (Mater Misericordiae University Hospital; MMUH) between October 2014 and September 2015 who were diagnosed with DVT. Patients presenting to the MMUH ED and diagnosed with DVT are managed according to a structured care pathway (under the governance of the multidisciplinary MMUH VTE Working Group). This pathway provides guidance for selection of patients suitable for outpatient DVT management. Clinical pre-test probability was assessed using the two level modified Wells Score. The Wells Score assigns points to clinical variables including active cancer treatment, paralysis, recent surgery, calf swelling and tenderness in order to predict likelihood of DVT [13]. In cases where DVT was unlikely (modified Wells score ≤ 1), D-dimer testing was carried out and a negative result out ruled DVT. In patients with ‘likely’ DVT (modified Wells score > 1) patients proceeded directly to imaging with compression ultrasonography (CUS). Patients with a confirmed diagnosis of DVT were managed as outpatients providing they did not meet the exclusion criteria listed in local guidelines: alcohol dependence, signs or symptoms suggestive of pulmonary embolus (PE) or confirmed PE, age < 18, patients already on anticoagulation at time of diagnosis, pregnancy, significant issues with compliance, cognition, mobility or communication, active or significant risk of bleeding, or comorbidities requiring medical or surgical admission (which at the time of the study included active malignancy, severe liver and renal impairment, bleeding disorder). Suitable patients preferring a long-term once daily option rather than twice daily therapy were prescribed rivaroxaban 15 mg twice daily for three weeks, followed by 20 mg once daily for three months total duration unless an indication to continue therapy existed upon review, including patients with unprovoked DVT or a persisting provoking factor. All other patients were prescribed therapeutic dose LMWH. Tinzaparin 175 units/kilogram once daily was the LMWH of choice at our centre. Patients were reviewed by the VTE clinical nurse specialist on the same day where possible. Patients for whom a clear date of discontinuation after three months was not suitable were followed up in the MMUH thrombosis clinic.

In this study, data were prospectively recorded at the time of diagnosis via the ED in a local hospital database. Variables recorded were age, Wells pre-test probability score, D-dimer, compression ultrasound result, treatment prescribed, presence of provoking factors, computed tomography pulmonary angiogram (CTPA) result (if also requested based upon symptomatology), previous VTE history, co-medications, early bleeding complications (specifically evaluated at 3 weeks post-diagnosis) and whether outpatient management was feasible. Follow up for sonographic evidence of residual thrombosis was not documented as part of this study.

Patients treated as per the outpatient VTE pathway were contacted by telephone by the VTE clinical nurse specialist at three days and three weeks post DVT to assess for early bleeding complications. This group of patients were scheduled for follow up at the coagulation outpatient clinic with the consultant haematologist three months post DVT at which time they were specifically asked about bleeding. There was no standardised methodology for data collection regarding early bleeding complications for patients that were admitted to hospital for treatment. The study relied on patients self-reporting episodes of bleeding in this cohort during the three month follow up.

We used Microsoft Excel for data entry. We used descriptive statistics to analyse our data. The categorical variables were reported in counts and percentages for our groups of interest; inpatients, outpatients and the total group. For the continuous variables, we calculated the mean accompanied by the standard deviation (SD) and the median accompanied by the interquartile range (IQR) for each group.

## Results

Fifty-one thousand five hundred forty-four patients presented to the MMUH ED during the study time period. Of these, 400 patients were investigated for DVT by the VTE clinical nurse specialist, and 78 (19%) had a confirmed diagnosis of DVT on ultrasound. Of the total number of those investigated, the diagnosis was ruled out with Wells Score and D-dimer in 63 (16%), while 259 (65%) had negative sonography.

Patient characteristics are outlined in Table 1. The majority of patients diagnosed with DVT were male (59%; 46/78). The mean age of the total cohort of patients was 47.7 years (SD 18.5). Most DVT events were associated with a provoking factor (63%; 49/78), in individuals without a previous history of VTE (64%; 50/78). Intravenous drug use was the provoking factor for 40% (31/78) of total patients and 63% (31/49) of provoked cases. Other provoking factors included immobilisation (20%; 10/49), oral contraceptive pill (6%; 3/49), post-partum (6%; 3/49) and cancer/active inflammation (4%; 2/49).

**Table 1:** Characteristics of Patients Presenting to the Emergency Department with DVT between 16/10/2014 and 02/09/2015

Patient Characteristics	Inpatient (*n* = 25)	Outpatient (*n* = 53)	Total (total *n* = 78)
Age			
Mean (SD)	48.1 (21.2)	47.8 (17.6)	47.7 (18.5)
Median (IQR)	40 (34.5)	43 (32)	41 (32.3)
Gender			
Male	13 (52%)	33 (62%)	46 (59%)
Female	12 (48%)	20 (38%)	32 (41%)
Wells Score:			
Unlikely	2 (8%)	6 (11%)	8 (10%)
Likely	10 (40%)	24 (45%)	34 (44%)
Not assessed	13 (52%)	23 (44%)	36 (46%)
D-dimer:			
Raised	12 (48%)	31 (58%)	43 (55%)
Normal Range	0 (0%)	2 (4%)	2 (3%)
Not measured	13 (52%)	20 (38%)	33 (42%)
History of VTE	9 (36%)	19 (36%)	28 (36%)
Provoking factor	17 (68%)	32 (60%)	49 (63%)
Associated PE	12 (48%)	3 (5%)	15 (19%)
Location:			
Upper limb	1 (4%)	1 (2%)	2 (3%)
Lower limb	24 (96%)	52 (98%)	76 (97%)
Treatment:			
Rivaroxaban	4 (16%)	31 (58%)	35 (45%)
LMWH	18 (72%)	22 (42%)	40 (51%)
Not recorded	3 (12%)	0 (0%)	3 (4%)
Medical Comorbidities	5 (25%)	17 (32%)	23 (55%)
IVDU	10 (40%)	21 (42%)	31 (39%)
Bleeding Complications	1 (4%)	5 (9%)	6 (8%)
Not recorded	23 (92%)	29 (54%)	52 (66%)
Post Partum	1 (4%)	2 (4%)	3 (4%)

In 54% (42/78) of cases, there was documentation of the clinical pre-test probability using the two level modified Wells score. In cases with documentation of Wells Score 19% (8/42) and 81% (34/42) had an unlikely probability and a likely probability of DVT respectively. Wells score was not documented in 46% of patients at presentation (36/78). For 58% (21/36) of these patients this was an appropriate omission of the score as individuals were post-partum or persons who inject drugs (PWID). 58% (45/78) of patients had a D-dimer level measured in the ED. In 51% (17/33) of patients without recorded D-dimer, the test was not indicated due to intravenous drug use. A D-dimer was ordered erroneously in three patients who presented post-partum.

Two (3%; 2/78) patients presented with upper limb DVT with the remainder of DVTs presenting at the level of the external iliac vein and below. 19% (15/78) of patients with DVTs had a coexisting PE at the time of presentation.

Sixty-eight percent (53/78) of patients were managed as outpatients while 32% (25/78) were admitted for treatment. 58% (31/53) of those treated as outpatients were treated with rivaroxaban while the remaining 42% (22/53) were treated with LMWH. 77% (17/22) of patients commenced on LMWH in the community in preference to rivaroxaban had a history of intravenous drug use. This treatment regimen is in accordance with the MMUH guidelines for outpatient DVT management. 70% (21/30) of PWID were managed with ambulatory care in the community. Of the total number of patients that were admitted to hospital 40% (10/25) were PWID. The documented reason for admission were as follows; 48% (12/25) had co existing pulmonary embolus, 12% (3/25) were admitted for thrombectomy, 16% (4/25) had psychosocial issues and 20% (5/25) had medical comorbidities requiring admission.

Bleeding complications were recorded in 9% (5/53) of those treated as outpatients versus 4% (1/25) of patients initially treated as inpatients. Reported bleeding complications included positive faecal occult blood, haemoptysis, haematuria, menorrhagia and bleeding from a groin abscess. There were a total of six cases of clinically relevant non major bleeding with no episodes of major bleeding recorded in either group assessed according to the International Society of Thrombosis and Haemostasis definitions [15]. In accordance with the outpatient DVT protocol, patients enrolled in the outpatient pathway and commenced on rivaroxaban received a follow up phone call at three days and three weeks from the VTE clinical nurse specialist (CNS) to assess bleeding complications. 47% (37/78) of patients in our study were followed up in this way. There were insufficient resources to formally follow up every patient included in the study with CNS led phone calls. Patients admitted to hospital for initial therapy were followed up by the admitting medical team, the outcomes of which were outside the scope of our data collection in certain cases. Patients anticoagulated with LMWH had contact with healthcare professionals in the majority of cases therefore follow up phone calls were not prioritised for this group for pragmatic reasons.

## Discussion

Implementation of an outpatient care pathway led to the majority of patients presenting with DVT in our institution being treated without hospital admission. Sixty-eight percent of all patients were treated as outpatients during the study period. Forty percent of patients with DVT were treated with a DOAC as an outpatient in line with international best practice guidelines. A further 28% were treated in the community with LMWH, 77% of those were PWID. There is a high prevalence of intravenous drug use among patients within the catchment of our inner city, urban centre. LMWH is currently recommended for pragmatic reasons for these patients, as most Dublin drug treatment centres are familiar with and most comfortable with direct administration of LMWH for these clients. With the development of pathways and infrastructure in inclusion health in Dublin city centre, this may change in the future. Inpatients were treated with LMWH (72%) or a DOAC (16%), with the remaining three cases undocumented. The most prominent barrier to ambulatory management in our cohort appears to be DVT with associated PE. Consequently, development of safe pathways for outpatient management of PE has been prioritised by the hospital VTE Committee. The implementation of validated risk assessment tools in the ED may identify low risk patients with PE suitable for outpatient management. The introduction of eligibility criteria and coordination with the outpatient department to ensure appropriate interval follow up for this cohort, could facilitate safe outpatient management of specific patients presenting with PE [16]. Almost 20% of patients presented with DVT and associated PE. This is similar to figures cited in international literature [11]. While the prevalence of intravenous drug use was stable between the inpatient and outpatient group (40% versus 42%) concomitant medical and psychosocial issues were documented in the group for admission. Development of inclusion health pathways in collaboration with medical social workers dedicated to the care and management of patients with social challenges including homelessness and drug and alcohol addictions has been prioritized as a key outcome of this study. In order to meet this objective, the Hospital VTE Committee will also work closely with colleagues in primary care who provide dedicated support to these vulnerable patients and with colleagues in other Dublin City Centre Hospitals, as patients with inclusion health needs frequently attend several hospitals due to their circumstances [17, 18].

In 34% of cases presenting to the ED where Wells Score was indicated (excluding PWID and post-partum cases), record of the score was inappropriately omitted from the patient documents. This is a crucial step in the evaluation of clinically suspected DVT and guides further investigation. This revealed non-compliance with the recommended structured care pathway for DVT in the ED and is not in line with best practice as outlined by international guidelines [13]. Patients with complete DVT work up including documentation of Wells Score were more likely to be treated as an outpatient. While D-dimer was measured in 58% of cases, deviation from clinical practice guidelines occurred in only one case when the test was inappropriately requested with a documented high clinical probability of DVT as established by the Wells Score [13]. The presence of a provoking factor and unusual site of DVT, such as upper limb thrombi, were both factors associated with admission to hospital.

The data in this study was collected consecutively and prospectively. All radiologically proven cases of DVT were recorded in the data set by the research team. This approach aimed to minimise bias due to low response and to prevent misclassification due to recall bias as can be seen in cross-sectional studies. The main limitation of the study was its observational design. Physicians in the ED were unaware that the data collection was taking place and therefore did not clearly document the clinical criteria supporting admission. Patients with suspected DVT were seen directly by the VTE CNS during office hours under the supervision of the Emergency Medicine (EM) consultant staff, but outside of these hours patients were assessed by junior and senior EM doctors, which likely accounts for variance in assessment and documentation. Contraindications to outpatient management were inferred from studying the patient’s clinical data in the context of the structured care pathway. This study reports the prevalence of DVT presenting to the ED and describes the subsequent management pathway, however it is not possible to fully elucidate the patient factors impacting clinical decision making and treatment choice. Cause and effect relationships and associations are be difficult to interpret. There were missing data in our study. Incomplete data entry was noted most particularly when recording early bleeding complications. Accurate records of follow up complications are not available to us for all cases. This study did not set out to primarily assess bleeding complications. We are limited in our ability to comment on bleeding complications between the two treatment pathways and between the different anticoagulant agents due to the lack of standardisation in our methods for assessing bleeding complications between the two groups. However, despite limitations, the study provides a clear picture of the clinical profile and management of patients in clinical practice.

## Conclusion

The results of this study are relevant throughout the world today. Clinical trials have long since established that outpatient DVT management is a safe and effective treatment. There is now an emerging evidence base for the outpatient treatment of pulmonary embolism [19]. However despite the progress in the field of research, it is clear from this data that there is scope for further development of the outpatient DVT pathway in clinical practice. The findings of this study are consistent when compared with international data [4, 12, 14]. Directing resources towards strategies which facilitate outpatient DVT treatment among vulnerable patient groups could represent a means of reducing hospital admissions for DVT in urban centres and, ultimately, lead to health care savings. The majority of hospitals in Ireland do not have a permanent VTE CNS in the ED. Due to the lack of resources and supported infrastructure it is difficult to effectively implement outpatient VTE management to its full potential. Our study highlights the success of this model, which should become standard as part of wider VTE care in Ireland.

## Data Availability

The datasets used and/or analysed during the current study are available from the corresponding author on reasonable request.

## Abbreviations

CNS: Clinical nurse specialist
CTPA: Computed tomography pulmonary angiogram
DOAC: Direct oral anticoagulant
DVT: Deep venous thrombosis
ED: Emergency Department
LMWH: Low molecular weight heparin
MMUH: Mater Misericordiae University Hospital
PE: Pulmonary embolism
PWID: Person who injects drugs
SD: Standard deviation
VTE: Venous thromboembolism

## References

[1] Thrombosis: a major contributor to the global disease burden (2014). Isth Steering Committee for World Thrombosis Day. J Thromb Haemost.

[2] Schulman S, Ageno W, Konstantinides SV (2017). Venous thromboembolism: past, Present and Future. Thromb Haemost.

[3] Raskob GE, Angchaisuksiri P, Blanco AN, Buller H, Gallus A, Hunt BJ, Hylek EM, Kakkar A, Konstantinides SV, McCumber M, Ozaki Y, Wendelboe A, Weitz JI (2014). Day ISCfWT. Thrombosis: a major contributor to global disease burden. Arterioscler Thromb Vasc Biol.

[4] Jimenez Sonia, Ruiz-Artacho Pedro, Merlo Marta, Suero Coral, Antolin Albert, Casal José Ramón, Sanchez Marta, Ortega-Duarte Alejandra, Genis Mar, Piñera Pascual (2017). Risk profile, management, and outcomes of patients with venous thromboembolism attended in Spanish Emergency Departments. Medicine.

[5] Vinson D, Berman D (2001). Outpatient treatement of deepvenous thrombosis: a clinical care pathway managed by the emergency department. Ann Emerg Med.

[6] Othieno R, Okpo E, Forster R (2018). Home versus in-patient treatment for deepvenous thrmobosis. Cochrane Database Syst Rev.

[7] Harrison Linda, McGinnis Joanne, Crowther Mark, Ginsberg Jeffrey, Hirsh Jack (1998). Assessment of Outpatient Treatment of Deep-Vein Thrombosis With Low-Molecular-Weight Heparin. Archives of Internal Medicine.

[8] Dunn AS, Schechter C, Gotlin A, Vomvolakis D, Jacobs E, Sacks HS, Coller B (2001). Outpatient treatment of deep venous thrombosis in diverse inner-city patients. Am J Med.

[9] Condliffe R (2016). Pathways for outpatient management of venous thromboembolism in a UK centre. Thromb J.

[10] Kearon C (2016). Antithrombotic Therapy for VTE Disease: CHEST Guideline and Expert Panel Report. Chest.

[11] Bauersachs R, Berkowitz SD, Brenner B (2010). Oral rivaroxaban for symptomatic venous thromboembolism. N Engl J Med.

[12] Imberti D, Barillari G (2017). Real life Management of Venous Thromboembolsim with rivaroxaban: results from experience VTE, an Italian epidemiological survey. Clinical and Applied Thrombosis/Haemostasis.

[13] Mazzolai L, et al. Diagnosis and management of acute deep vein thrombosis: a joint consensus document from the European society of cardiology working groups of aorta and peripheral circulation and pulmonary circulation and right ventricular function. Eur Heart J. 2018;39(47):4208–18. 10.1093/eurheartj/ehx003 28329262

[14] Douce D (2017). Outpatient Tretament of DeepVein thrombosis in the United States: the reasons for Geogrphical and racial differences in stroke study. J Hosp Med.

[15] Kaatz S, Ahmad D, Spyropoulos AC, Schulman S (2015). Subcommittee on control of anticoagulation.. Definition of clinically relevant non-major bleeding in studies of anticoagulants in atrial fibrillation and venous thromboembolic disease in non-surgical patients: communication from the SSC of the ISTH. J Thromb Haemost.

[16] Roy P, Moumneh T, Penaloza A, Sanchez O (2017). Outpatient management of pulmonary embolism. Thromb Res.

[17] Our Health Service - Inclusion Health Service At St James's Hospital. Health Servicec Executive. URL: https://www.hse.ie/eng/about/our-health-service/making-it-better/inclusion-health-service-at-st-james-hospital.html. Accessed at: 5 May 2018.

[18] Safety net primary care. URL: https://www.primarycaresafetynet.ie/. Acccessed 5 May 2018.

[19] Bledsoe JR, Woller SC, Stevens SM, Aston V, Patten R, Allen T, Horne BD, Dong L, Lloyd J, Snow G, Madsen T, Elliott CG. Management of low-Risk Pulmonary Embolism Patients without Hospitalization: the low-risk pulmonary embolism prospective management study. Chest. 2018;(18)30231–9. 10.1016/j.chest.2018.01.035 29410163

